# Gli1 promotes the phenotypic transformation of valve interstitial cells through Hedgehog pathway activation exacerbating calcific aortic valve disease

**DOI:** 10.7150/ijbs.74123

**Published:** 2023-04-09

**Authors:** Yuming Huang, Chen Jiang, Liang Chen, Juanjuan Han, Ming Liu, Tingwen Zhou, Nianguo Dong, Kang Xu

**Affiliations:** 1 Department of Thoracic Surgery, The First Affiliated Hospital of Nanjing Medical University, Nanjing 210029, China.; 2 Department of Cardiovascular Surgery, Union Hospital, Tongji Medical College, Huazhong University of Science and Technology, Wuhan 430022, China.; 3 Hubei Engineering Technology Research Center of Chinese Materia Medica Processing, College of Pharmacy, Hubei University of Chinese Medicine, Wuhan 430065, China.

**Keywords:** Calcific aortic valve disease (CAVD), glioma-associated oncogene 1 (Gli1), Hedgehog signaling pathway, AKT signaling pathway, osteogenic transformation

## Abstract

Calcific aortic valve disease (CAVD) is the most prevalent human valve disease worldwide. Multiple factors induce "irreversible" pathological changes in the aortic valve leaflets, resulting in changes in cardiac hemodynamics, eventually leading to heart failure. However, no effective pharmaceutical interventions have been found and prosthetic valve replacement is the only curative approach. Glioma-associated oncogene 1 (Gli1) exerts a regulatory role on cardiovascular diseases, and it is already a therapeutic target to combat tumors. Our research aimed to explore the role and basic mechanism of Gli1 in CAVD, to pave the way for the discovery of effective drugs in the treatment of CAVD. Human aortic valve tissues were obtained to evaluate Gli1 expression and primary valve interstitial cells (VICs) were used to perform related experiments. The results showed that Gli1 promoted cell proliferation and significantly accelerated cell osteogenic transformation through the up-regulation of the osteogenic factors Runx2 and Alp, in turn through the AKT signaling pathway by targeting P130cas expression. Furthermore, Gli1 was activated by TGF-β and sonic hedgehog through the canonical and non-canonical Hedgehog signaling pathways in VICs. Our results indicated that Gli1 promoted cell proliferation and accelerated cell osteogenic transformation in VICs, providing a new strategy for the therapy of CAVD by targeting Gli1.

## Introduction

Calcified aortic valve disease (CAVD) is, by far, the most prevalent human valve disease worldwide. Although its prevalence is only 0.2% in the middle-aged people, it increases to 9.8% in octogenarians 1-3. CAVD is characterized by a slow progressive fibro-calcific remodeling of the valve leaflets. As a consequence of that, hemodynamic changes happen in the cardiovascular system contributing to heart failure 4. So far, no effective pharmaceutical interventions have been found to stop or just slow down the progression of CAVD; thus when this disease reaches the “point of no return”, only aortic valve replacement is the solution to cure 5. Recently, the use of transcatheter aortic valve replacement therapy has increased. However, despite the good results achieved, the way to success is still far. Therefore, it is of utmost importance to explore the mechanisms regulating CAVD initiation and progression to identify promising therapeutic targets.

The transcription factor glioma-associated oncogene 1 (Gli1) has been recognized as a key nuclear executor at the distal end of the hedgehog signaling pathway. It plays a crucial role in regulating many biological processes, such as differentiation, proliferation, and apoptosis 6. Previous studies on the hedgehog pathway mostly focus on the embryonic development and neural tissue homeostasis, or cancer stemness, but its regulatory role in cardiovascular disease has been continually ignored 7. One research group in Germany identified the hedgehog signaling pathway as a specific process for activating adventitial fibroblasts during vasculogenesis, and promoting angiogenesis in the adult 8. Another group previously found that the activation of the hedgehog pathway induces the proliferation of vascular smooth muscle cells during intimal hyperplasia 9. French researchers also found that the hedgehog pathway plays an important role in cardio-protection against ischemia-reperfusion injuries 10. More functions of the Gli1 molecule in cardiovascular diseases have been explored.

The pathogenesis of CAVD is now considered a complex pathophysiological process involving network modulation between multiple cells and multiple mechanisms. The predisposing and causative mechanisms include: endothelial dysfunction, lipid accumulation, sterile inflammation, excessive production or degradation of the extracellular matrix, excessive proliferation of immune cells, myofibroblastic/osteoblastic differentiation of valvular interstitial cells (VICs) and subsequent calcification 11-13. Therefore, our hypothesis is that Gli1 inevitably plays an important role in this disease due to the above characteristics of the valvular calcification pathological process and the role of the hedgehog signaling pathway found in cardiovascular disease. Gli1 might function to induce VICs proliferation and accelerate VIC calcification. The present research showed for the first time that hedgehog signaling pathway was activated during CAVD. The target factor Gli1 induced the proliferation of VICs and promoted the expression of Runx2 as the key factor in calcification.

This work aimed to investigate the role of Gli1 in CAVD disease. Our results indicated that Gli1 significantly promoted the calcification of VICs and accelerated their proliferation. Furthermore, Gli1-AKT-P130cas axis played an important role in the osteogenic differentiation of VICs. The mechanism of Gli1 activation was also explored, revealing that both canonical and non-canonical Hedgehog signaling pathways were involved in the upregulation of the Gli1 transcription factor in VICs. Thus, the inhibition of the expression of Gli1 might be a new and effective strategy for delaying the osteogenic differentiation of human aortic VICs.

## Materials and Methods

### VIC isolation and culture

Human aortic valves were obtained from patients undergoing heart valve surgery at the cardiovascular surgeon department of the Union hospital. Patients provided a written informed consent, and the study was approved by the Ethics Committee of Tongji Medical College, Huazhong University of Science and Technology in Wuhan, China. Aortic valve leaflets were excised and rinsed according to our previous protocol. Then tissues were minced and placed in collagenase (150 units/mL) in Dulbecco's Modified Eagle's Medium (Hyclone, Logan, UT, USA) for 6-8 h at 37 °C. The cell suspension was obtained at the end of collagenase digestion by removing the undigested tissue with a 70 μm cell strainer. The cells were cultured in standard DMEM supplemented with 10% heat-inactivated FBS (Thermo Fisher Scientific, Waltham, MA, USA) and 150 U/mL penicillin/streptomycin (Hyclone). VICs were seeded at a density of 10,000 cells/cm^2^ in tissue culture flasks in a complete medium, which was changed every 3 days until VICs reached 90% confluence. Cells at the third or fourth passage were used in all the experiments.

### Adenovirus plasmid overexpression vehicles, Small-interfering RNA and cell transfection, and small molecule inhibitors

The overexpression adenovirus plasmid of GLI family zinc finger 1 (GLI1) and control adenovirus were purchased from Vigene Biosciences (Shandong, China). Valve interstitial cells were transfected with small-interfering RNA (Ribobio bio, Guangzhou, China) targeting SMAD2, SM AD3, SMAD4, SHH, GLI1, BCAR1, using lipofectamine 3000 kits (Invitrogen, Carlsbad, CA, USA) according to the manufacturer's instructions after cells were grown to 60% confluence. All small molecule inhibitors were bought from Selleck Chemicals (Houston, TX, USA): Gli1 selective inhibitor GANT61 (S8075), AKT1 selective inhibitor MK-2206 (S1078), TGF-β type I receptor-selective inhibitor SB431542 (S1067), PTCH1 selective inhibitor GDC-0449(S1082).

### RNA extraction and qPCR analysis

Total RNA was extracted with Trizol reagent (Invitrogen, Carlsbad, CA) and the reverse transcription product was used as a template to perform real-time polymerase chain reaction (PCR) on a StepOne Plus thermal cycler (Applied Biosystems, Foster City, CA) using a 2x SYBR Green qPCR Master Mix (High ROX) (Bimake, Houston, TX) following the manufacturer's guide. All the primers were referenced from the previous study, and primer sequences were shown in Supplementary Table 1. The final data were analyzed by the 2^-ΔΔct^ method 14-16.

### Western blotting

For western blotting, cell samples were extracted and quantified then boiled at 95℃, 5min. The protein sample was separated on an 8% sodium dodecyl sulfate-polyacrylamide gel electrophoresis gel then transferred on a polyvinylidene fluoride membrane. Incubating primary antibodies overnight at 4℃, followed by the corresponding secondary antibodies for protein expression visualization. Primary antibodies were shown in Supplementary Table 2 17.

### Immunofluorescence Staining

**For tissue:** Tissues were fixed in 4% paraformaldehyde overnight, then dehydrated by 15% and 30% sucrose solution. Six microns frozen sections of formalin-fixed human valve tissues were cut and used for staining.

**For cell:** VICs seeded into 24-well plates at a density of 10000 cells/well were washed twice with PBS and fixed in 4% paraformaldehyde for 15 min. The fixative solution was removed by rinsing three times with PBS. Cells were permeabilized with 0.2% Triton X-100 for 10 min, washed three times with PBS, and blocked for 30 min with goat serum albumin (Boster, Wuhan, China). Immediately after blocking, cells were incubated with primary antibodies at 4°C overnight. After washing three times with PBS, samples were incubated with secondary antibodies (CST) in PBS for 60 min at room temperature. Then samples were washed twice with PBS and incubated with DAPI (Biofroxx GmbH, Einhausen, Germany) for 4 min to stain the nuclei. Samples were washed twice with PBS and then imaged on the Axio Observer Z1 microscope (Zeiss, Oberkochen, Germany) 18.

### Chromatin Immunoprecipitation (ChIP) and ChIP-seq analysis

Cells were cross-linked with 1% formaldehyde for 10 min at room temperature and the reaction was subsequently stopped by adding glycine to a final concentration of 0.125 M for another 10 min at room temperature. Then, the cells were washed twice in cold PBS and harvested in lysis buffer (1% Triton X-100, 0.1% SDS, 150 mM NaCl, 1 mM EDTA, 20 mM Tris, pH 8.0, and complete protease inhibitor mixture). The samples were sonicated 20 times (30 s on/off, 260 W) at 4 °C using a Diagenode Bioruptor. After pre-clearing overnight, samples were incubated with IgG (sigma, #R2655) previously conjugated with Dynabeads Protein G (Invitrogen, #10009D) for 3 h at 4 °C, and the immunocomplexes were washed twice with buffer 1 (20 mM Tris-HCl pH 8.0, 2 mM EDTA, 150 mM NaCl, 0.1% SDS, 1% Triton X-100), once with buffer 2 (20 mM Tris-HCl pH 8.0, 2 mM EDTA, 500 mM NaCl, 0.1% SDS, 1% Triton X-100), once with buffer 3 (10 mM Tris-HCl pH 8.0, 1 mM EDTA, 250 mM LiCl, 1% Sodium deoxycholate, 1% NP-40), and twice with buffer 4 (10 mM Tris-HCl pH 8.0, 1 mM EDTA). Each wash was performed for 5 min at 4 °C rotation. DNA-protein complexes were eluted with 200 µl elution buffer (1% SDS and 0.1 M NaHCO3) and de-cross-linked by adding 0.2 M NaCl and shaking overnight at 65 °C. Then, digestion was performed using proteinase K, and the enriched DNA was purified by QIAquick PCR purification kit (Qiagen, #28104). The ChIP DNA libraries of VICs were sequenced on an Illumina HisSeq-3000. Reads were aligned to Ensembl human genome (hg19) using the Bowtie software. After format conversion and sorting, duplicate reads were removed by the rod up tool from the SAMtools package. The mapped sequence reads were processed using MACS version 1.4.2 against their matching control samples to identify the significant binding site of Gli1, and only peaks with *P* values < 10^-5^ were kept for further analyses. A Perl script annotatePeaks.pl in the HOMER package was used to associate ChIP peaks with nearby genes, and the visualization of the location and the shape of the called peaks was performed using Integrative Genomics Viewer (IGV).

### FACS for cell cycle and Cell Viability Assay

VICs (passage 3) were cultured in 60 mm dishes until 80% confluency. Then, the medium was changed to DMEM with 2% FBS for 8 h before performing the different treatments. The samples were treated with trypsin and re-suspended in PBS at a density of 5×10^5^/mL, followed by fixation in 70% precooled ethanol overnight at 4 °C, centrifugation, washing, and staining with PI/RNase staining buffer (BD Biosciences) for 30 min at 4 °C. Cell count at different phases of the cell cycle was performed using FCM as previously described 19.

### Cell Viability Assay

Cell viability was assessed with the Cell Counting Kit-8 (CCK-8) assay (Bimake.com, Houston, TX, USA) according to the manufacturer's instructions. The cells were seeded at a density of 5000 cells/well in 24-well plates and cultured for 1-6 days. At the end of each time interval, cell samples were washed with PBS and incubated with a serum-free medium containing 10% CCK-8 reagent. After 4 h of incubation at 37°C in an atmosphere of 5% CO2, aliquots were pipetted into a 96-well plate and measured at 450 nm using an enzyme-labeling instrument (Thermo Fisher Scientific) 20.

### Edu assay

Cell proliferation ability was tested by Edu assay (Ribobio bio, Guangzhou, China) according to the manufacturer's instructions. The cells were seeded at a density of 10000 cells/well in 12-well plates. After different treatments, VICs were cultured in a medium containing Edu for 2h at 37°C in an atmosphere of 5% CO2. The cell samples were fixed by 4% paraformaldehyde for 15 min then washed several times, stained with apollo staining solution for 30min, observed under the fluorescent microscope 21.

### Calcification analysis

Cells were seeded into 12-well plates and grown for three days until 90% confluency and further cultured in an osteogenic differentiation medium (OM) containing 10mM β-glycerophosphate, 100nM dexamethasone, 50μg/ml vitamin C, 1% FBS, 100 IU/ml penicillin/streptomycin under different culture conditions for 15 days. The degree of cell calcification was measured using Alizarin Red S staining. In brief, cells were incubated in ddH20 and the amount of Alizarin Res S dye released from the extracellular matrix was measured at 450 nm using an enzyme labeling instrument 22. For ex vivo osteogenic differentiation, human aortic valve leaflets were harvested from patients undergoing Bentall surgery due to acute type I aortic dissection. (or can be heart transplantation samples, should be normal via visibility). The samples (avoid the marginal area) were cut into 1mm*1mm (or can be bigger, no more than 1 cm*1cm) small pieces and treated with OM culture medium for at least 21 days. (Note: the small pieces were floaty culturing without attachment to the culture dishes). After OM induction, the small pieces can be transfected with re-comb adenovirus or chemical factors for one to two months (The treated time should be tested according to specific condition, no more than two months). The small pieces were fixed with 4% PFA for 30 min, then embedded in paraffin and 8-μm-thick sections were made. The specimens were stained with hematoxylin and eosin (H&E), Von kossa, and histological immunofluorescence staining for specific antibodies.

### RNA sequencing of VICs

RNA sequencing (RNA-seq) was utilized to compare the mRNA profiles between different treatments for VICs. Isolated RNA was sent to BGI Tech Solutions Co., Ltd. (Shenzhen, China) for RNA-seq, which was performed on the BGISEQ-500 sequencer; all samples were sequenced in triplicate for confirmation purposes. Sequencing results were analyzed using the “R Project (version 3.5.1)” to identify differentially expressed genes (DEGs). Gene Ontology (GO) and Kyoto Encyclopedia of Genes and Genomes (KEGG) pathway enrichment analyses were also performed 23.

### Statistical analysis

RNA-seq results were analyzed using the R (version 3.5.1) according to a previous study and all other data were analyzed and expressed as the mean ± standard deviation (SD). Statistical comparisons were made by analysis of variance to evaluate differences among groups. A p-value less than 0.05 was considered statistically significant.

## Results

### Increased expression of Gli1 in human calcific aortic valve tissues

**Figure 1:**
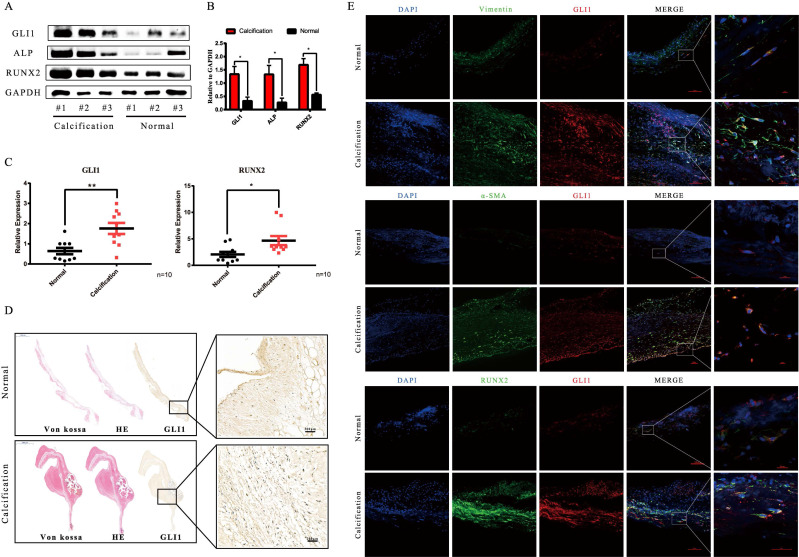
Analysis of Gli1 expression in aortic valve tissues from CAVD and healthy samples. (A) and (B) western blotting analysis showed that Gli1, Runx2, ALP proteins increased significantly in CAVD samples compared with normal ones. (C) RT-PCR was applied to detect the mRNA expression level of Gli1 and Runx2 in 10 pairs of aortic valve samples from CAVD and normal patients. Data are expressed as the mean ± SD, n=10. Data were analyzed using one-way ANOVA, (*) *p* < 0.05 indicates a significant difference. (D) HE, Von Kossa staining along with Gli1 Immunohistochemical staining were performed in calcified aortic valves and normal ones. (E) Dual immunofluorescence staining of Gli1, α-SMA (α-smooth muscle actin), Vimentin, Runx2, and DAPI were performed in calcified aortic valves and normal ones.

Tissues from 20 pairs of calcific aortic valves and adjacent normal tissues were used to analyze Gli1 expression in CAVD conditions. Gli1 significantly increased in calcific valves as well as Runt-related transcription factor 2 (Runx2) and Alkaline Phosphatase (ALP) which are markers of osteogenesis, as demonstrated by western blot** (Fig. 1A and Fig. 1B)**. The qRT-PCR results were consistent with those of protein expression **(Fig. 1C)**. HE, Von Kossa staining along with Gli1 immunohistochemical staining showed the presence of calcification nodules in calcified aortic valves and Gli1 was high expressed in the lesions **(Fig. 1D).** Dual immunofluorescence staining demonstrated that Gli1 was colocalized with α-smooth muscle actin (α-SMA), Vimentin, Runx2 in calcified aortic valves **(Fig. 1E)**. These results suggested that Gli1 accumulation in aortic valves might play a role in VIC phenotypic transition and aortic valve calcification.

### Total RNA-seq analysis after the regulation of Gli1 expression in valve interstitial cells

**Figure 2:**
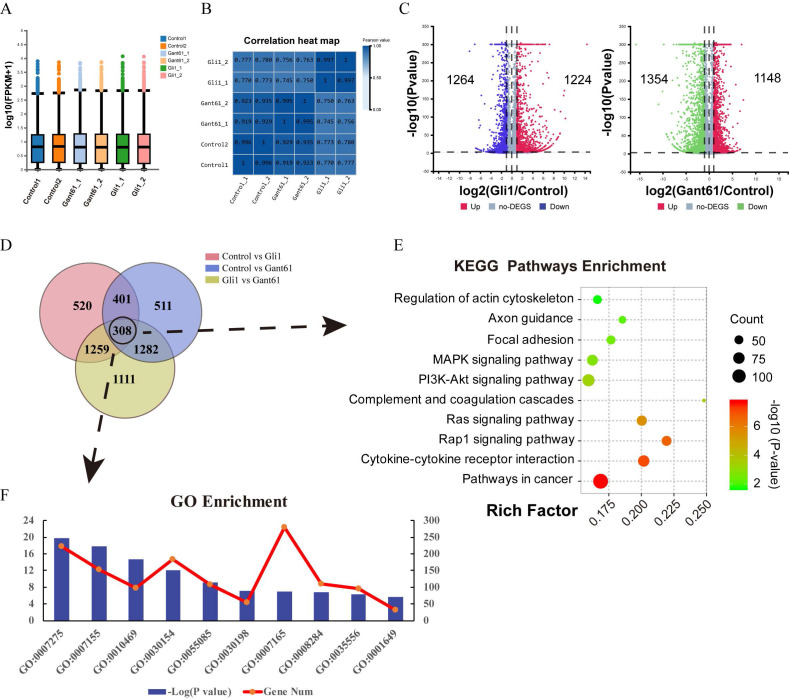
Total RNA-seq analysis after regulating the expression of Gli1 in VICs. (A) and (B) The box plot and correlation heat map the in the control group, Gli1 group, and Gant61 group. (C) volcano map of differentially expressed genes (DEGs) in these three different groups. Gli1 group versus control group: up-regulation 1224 genes and down-regulation 1264 genes; Gant61 group versus control group: up-regulation 1148 genes and down-regulation 1354 genes. FC (fold change) > 1 was accepted as positive DEGs. (D) Venn interaction of DEGs of the three groups compared with each other and 308 common DEGs were found. (E) KEGG pathway enrichment of common DEGs, bubble colors (deep) indicate the degree of enrichment (-Log10(P-value)), bubble size indicates gene counts matched the pathway enrichment, and rich factor indicates the matched gene counts in the integrated pathway background genes. (F) GO biological function enrichment of common DEGs, blue bars represent the degree of enrichment (-Log10(P-value)), red polyline showed the gene numbers involved.

Gant61, a selective Gli1 inhibitor that inhibits the DNA binding function of Gli1, was used to block the function of Gli1. An Adenovirus containing Gli1 expression cassette was used to induce Gli1 factor overexpression. The box plot and correlation heat map showed the distribution of the gene expression among different groups. Gli1 enrichment group was differently scattered compared with the Gant61 group and control group **(Fig. 2A and Fig. 2B)**. When Gli1 was overexpressed, 1224 genes were upregulated while 1264 genes were down-regulated (P<0.05). When Gli1 was blocked by Gant61, 1148 genes were upregulated while 1354 genes were down-regulated (P<0.05)** (Fig. 2C)**. The Venn diagram of DEGs in the Gli1 group *versus* control, Gant61 group *versus* control, and Gli1 group *versus* Gant61 group, revealed the presence of 308 differentially expressed genes (DEGs), which were used for further analysis** (Fig. 2D)**. All these DEGs were analyzed by the Kyoto Encyclopedia of Genes and Genomes (KEGG) pathway enrichment analysis and GO function annotation** (Fig. 2E and Fig. 2F)**. The top ten significant pathways and biological functions were listed. Genes related to cell differentiation, inflammatory response, extracellular matrix organization, and positive regulation of cell proliferation were modified.

### Gli1 promoted the proliferation of VICs and regulated the cell cycle of VICs

**Figure 3:**
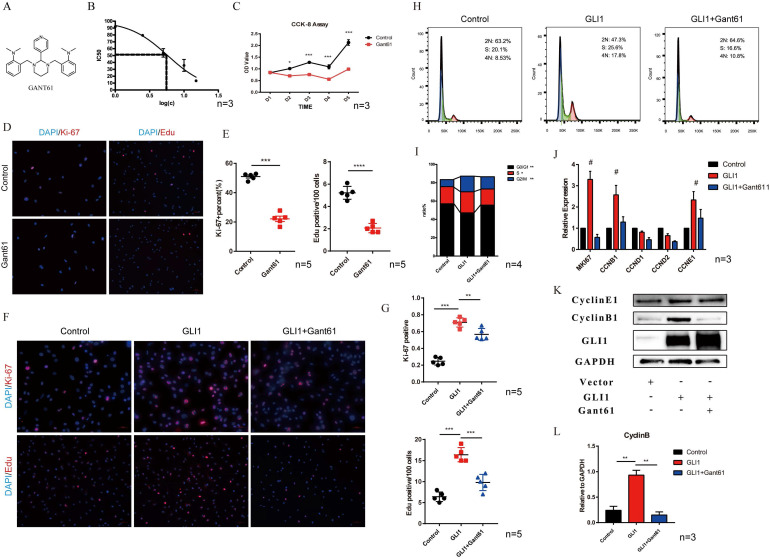
Gli1 promotes the proliferation of VICs and regulates the cell cycle. (A) Molecular structure of Gant61. (B) IC50 of Gant61 on VICs, concentrations were transferred to Log(c). (C) CCK-8 assay was performed in the control group and Gant61 group. (D) Ki-67 and Edu staining in VICs were performed in both control and Gant61 groups. (E) Semi-quantitative statistics on the percentage of Ki-67 positive cells and Edu positive cells. (F) Ki-67 and Edu staining in VICs under different treatments: control, Gli1, and Gli1+Gant61. (G) Semi-quantitative statistics on the percentage of Ki-67 positive cells and Edu positive cells. (H) and (I) Flow cytometry cell cycle of VICs in a different group and semi-quantitative statistics of each phase ratio. (J) and (K) RT-PCR and western blotting analysis of cell cycle-related factors in different groups. (L) Semi-quantitative statistics on the cyclin B protein. Data are expressed as the mean ± SD. Data were analyzed using one-way ANOVA, (*) *p* < 0.05 indicates a significant difference.

The molecular structure of Gant61 is presented in **Fig. 3A**. IC50 was calculated to assess the toxicity of Gant61 on VICs, and the result indicated that Gant61 showed clear signs of toxicity at concentrations above 5 μM** (Fig. 3B)**. Therefore, all subsequent *in vitro* experiments were performed using 5 μM Gant61. CCK-8 assay, ki-67, and Edu staining were performed to analyze the proliferation of VICs under Gli1 treatment with or without Gant61. Cell growth was almost completely inhabited when incubated in the presence of Gant61 **(Fig. 3C)**. Ki-67 and Edu staining indicated that the proliferation of VICs was reduced **(Fig. 3D)**. The statistical analysis showed that the proportion of positive cells was significantly different when performed with and without Gant61 **(Fig. 3E)**. In addition, the overexpression of Gli1 in VICs rescued the inhibition of cell proliferation in the presence of Gant61, Ki-67 and Edu staining showed that the proportion of positive cells in the Gli1 group was the highest among the three groups and statistically significant **(Fig. 3F and Fig. 3G)**. The cell cycle of VICs by flow cytometry showed that the treatment with Gli1 alone resulted in a unnormal cell growth **(Fig. 3H)**. Our results indicated that the proportion of cells at the G0/G1 phase and S phase was significantly increased **(Fig. 3I)**. qRT-PCR demonstrated that MKI67, CCNB1, CCNE1, and cell cycle-related genes, were significantly increased **(Fig. 3J)**. Western blotting showed consistent results, since cyclinB1 protein significantly increased in the group with Gli1 overexpression and this effect was blocked by the Gli1 inhibitor Gant61** (Fig. 3K and Fig. 3L)**.

### Gli1 accelerated the osteogenic differentiation of VICs

**Figure 4:**
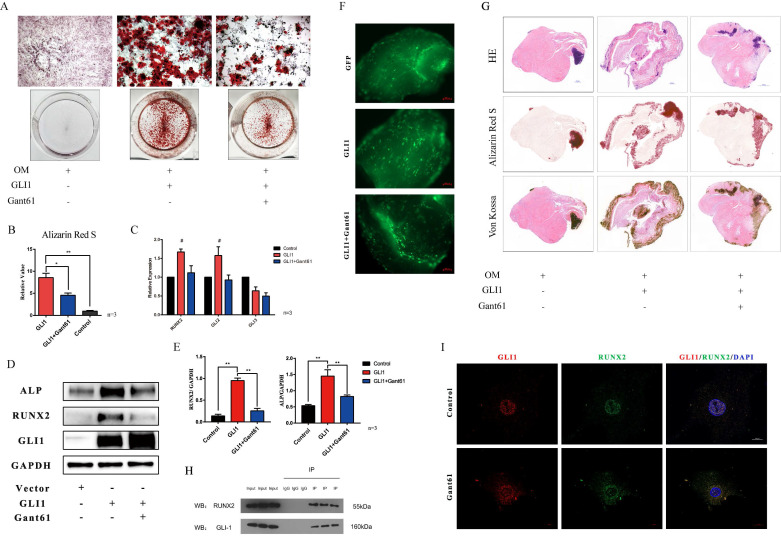
Gli1 accelerates the osteogenic differentiation of VICs. (A) Alizarin Red S staining of the cells under different conditioned cultures. (B) Semi-quantitative statistics of Alizarin Red S stain. (C) RT-PCR of Runx2, Gli2, Gli3 for VICs stimulated with Gli1 and then treated with Gant61 or not for 48h. Data were analyzed using one-way ANOVA, (#) versus control, p < 0.05 indicates a significant difference. (D) and (E) Western blotting and semi-quantification of ALP, Runx2 for VICs stimulated with Gli1 and then treated with Gant61 or not for 72h. Data were analyzed using one-way ANOVA, (*) p < 0.05 indicates a significant difference. (F) and (G) Ex-vivo osteogenic differentiation of aortic valve tissues in different conditions. Observed under the fluorescent microscope and Representative HE, Alizarin Red S, Von Kossa staining figures of each tissue. (H) and (I) Co-IP and cellular immunofluorescence staining of Gli1 and Runx2 proteins in VICs.

The osteogenic differentiation medium (OM) was used *in vitro* to induce the calcification of VICs. In addition, cells with Gli1 overexpression alone or combined with Gant61 were subjected to Alizarin Red S staining to evaluate the formation of the calcified nodules. Gli1 treatment group and Gli1+Gant61 group showed a positive staining and were significantly different than the control group** (Fig. 4A)**. The semi-quantification of calcification demonstrated a more than five times increase in the Gli1 group compared to the control group, while the calcification in the Gli1+Gant61 group was half that in the control **(Fig. 4B)**. Runx2 and Gli family genes Gli2, Gli3 were measured in the three groups Gli1, Gli1+Gant61, and Control. Our results showed that Runx2 increased more than 1.5-fold in the Gli1 group compared with the control group, and similar result was observed for Gli2 gene expression, indicating a synergy with Gli1, while Gli3 which works opposite to Gli1, was decreased in the Gli1 group **(Fig. 4C)**. Next, Runx2 and ALP protein expression was measured after the treatment with Gli1 overexpression for 72 h. The results indicated that the osteogenesis-related proteins Runx2 and ALP significantly increased in the Gli1 group** (Fig. 4D and Fig. 4E)**. Moreover, an *ex-vivo* osteogenic differentiation model was performed. Human aortic valve tissues were cut into small pieces and cultured in OM conditions. The tissues were subjected to Gli1 overexpression alone by transfection with adenovirus or combined with Gant61 treatment for two months** (Fig. 4F)**. HE, Von Kossa, and Alizarin Red S staining showed a significant difference between the three groups **(Fig. 4G)**. In addition, Gli1 and Runx2 interacted with each other as revealed by co-immunoprecipitation (Co-IP) experiments **(Fig. 4H)**. Cellular immunofluorescence staining showed that Gli1 and Runx2 were co-localized in the control group and Gant61 group** (Fig. 4I)**.

### Identification of Gli1 candidate target genes related to osteogenic differentiation

**Figure 5:**
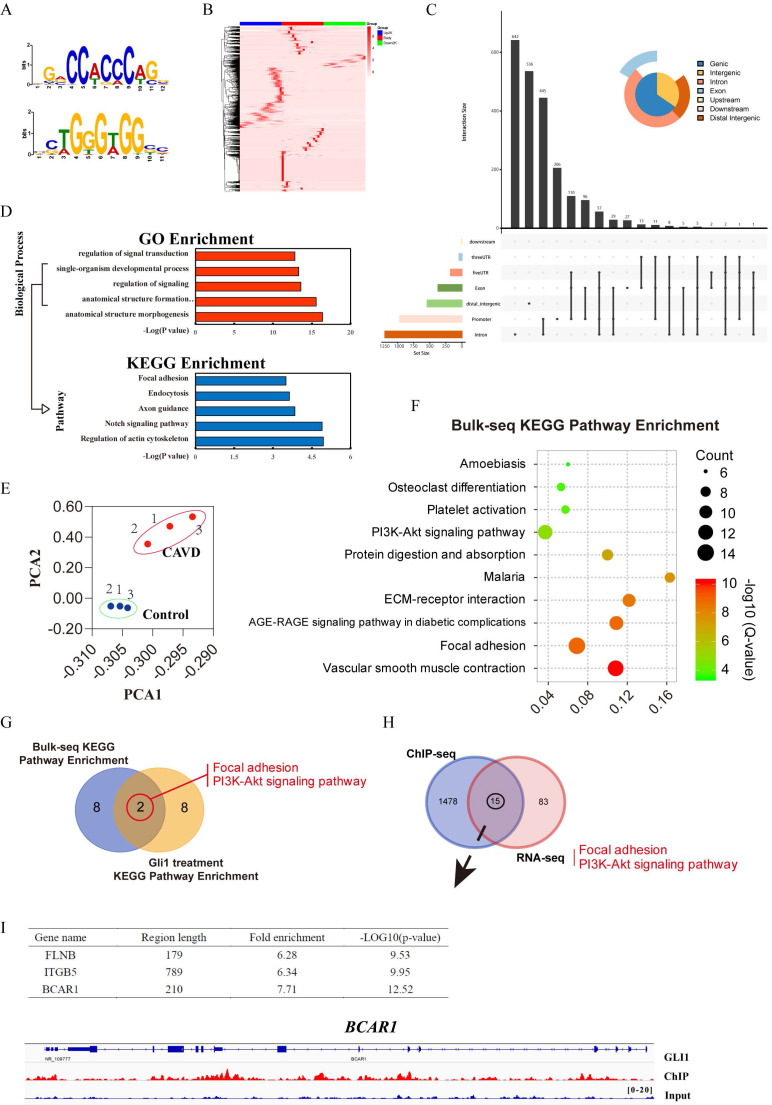
Identification of Gli1 candidate target genes related to osteogenic differentiation. (A) and (B) The TF binding motifs of Gli1 and the heatmap of the Gli1 binding site within 2kb from the transcriptional starting site. (C) Peak calling analysis of Gli1. (D) GO biological process enrichment and KEGG pathway enrichment analysis for Gli1 binding genes. (E) Principal Component Analysis (PCA) analysis for three pairs of aortic valves. (F) KEGG signaling pathway enrichment analysis of DEGs between samples from the CAVD group and the control group. (G) Venn's interaction of KEGG signaling pathway enrichment analysis between tissue RNA-seq and cellular RNA-seq. (H) Venn's interaction of the two common pathways with ChIP-seq data. (I) Fold enrichment and p-value of the final target genes according to the ChIP-seq data.

Three sets of sequencing data were used for comprehensive analysis to explore the possible targets of Gli1 to induce calcification of VICs: RNA-sequencing data of Gli1-treated cells, ChIP-sequencing data of Gli1 in VICs, and RNA-sequencing data of different valve tissues. The TF binding motifs of Gli1 in VICs were CCACCC and TGGGTGG **(Fig. 5A)** and the Gli1 binding sites within 2kb from the transcriptional starting site (TSS) were evaluated to discover the candidate genes **(Fig. 5B)**. Peak calling analysis of Gli1 analyzed peak positions relative to gene locations and revealed the distribution of the binding sites **(Fig. 5C)**. GO biological process enrichment and KEGG pathway enrichment analysis showed the top five significant processes and pathways for Gli1 binding genes** (Fig. 5D)**. Three pairs of aortic valves were chosen to perform RNA-seq. The entire transcriptome profile of these samples was performed by Principal Component Analysis (PCA). The results showed that the calcific valves and the normal ones were clustered in their respective groups **(Fig. 5E)**. KEGG signaling pathway enrichment analysis showed the top ten significant pathways **(Fig. 5F)**. After Venn's interaction of the top ten significant activated signaling pathways between tissue RNA-seq and cellular RNA-seq, two common pathways were identified: focal adhesion and PI3K-AKT signaling pathway** (Fig. 5G)**. The Venn plot showed 15 candidate genes that may play a critical role in calcification after integration of the DEGs related to the two common pathways PI3K-AKT signaling and focal adhesion with ChIP-seq data** (Fig. 5H)**. Finally, three potential direct binding genes such as FLNB, ITGB5, and BCAR1 were chosen according to the fold enrichment and *p*-value **(Fig. 5I)**. BCAR1 was selected as the final target gene which encodes the protein P130cas. The high-resolution peak identified from the reading density of the ChIP-Seq data revealed a binding region in the BCAR1 gene **(Fig. 5I)**.

### Gli1-P130cas-AKT axis promoted the osteogenic differentiation of VICs

**Figure 6:**
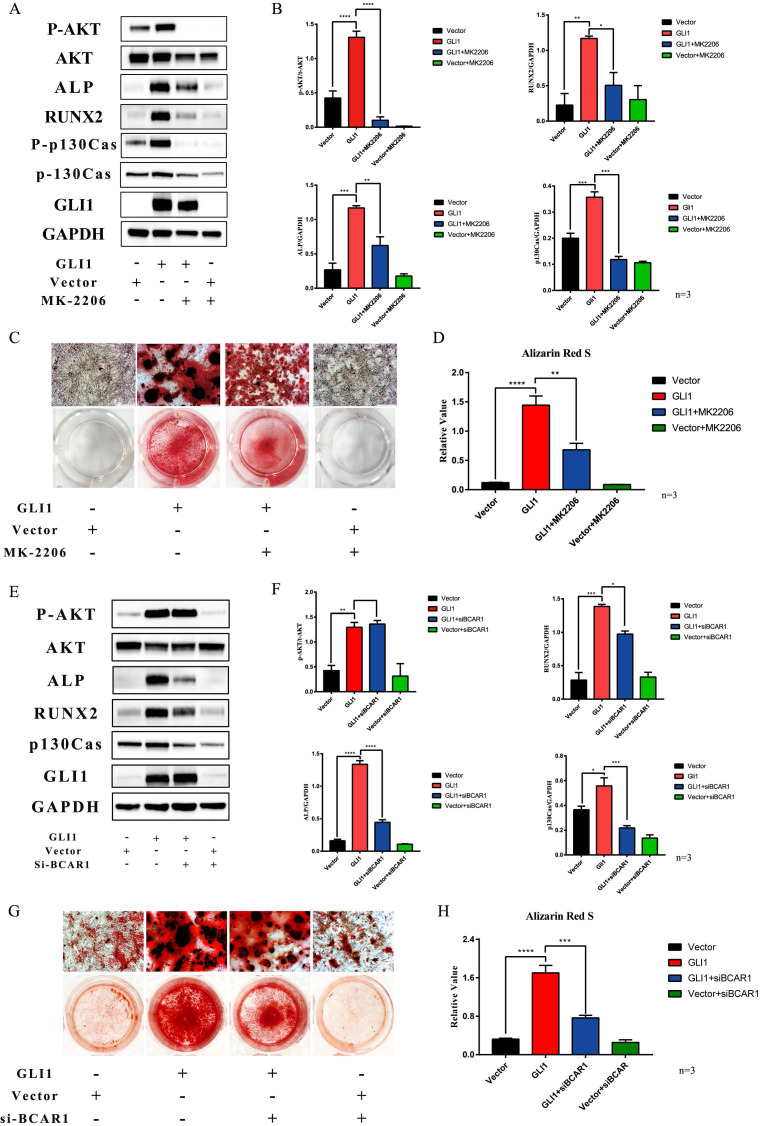
Gli1-P130cas-AKT axis promotes the osteogenic differentiation of VICs. (A) Western blotting for p-AKT, AKT, ALP, p-p130cas, p130cas under the Gli1 treatment with or without MK2206. (B) Statistical analysis of Western Blotting gray values of p-AKT/AKT, p-p130cas/p130cas, ALP, and RUNX2. Data were analyzed using one-way ANOVA, (*) p < 0.05 indicates a significant difference. (C) Calcification nodules were tested by Alizarin Red S after being cultured under different treatments. (D) Semi-quantitation of calcification. Data were analyzed using one-way ANOVA, (*) p < 0.05 indicates a significant difference. (E) Western blotting for p-AKT, AKT, ALP, p-p130cas, p130cas under the Gli1 treatment with or without si-BCAR treatment. (F) Statistical analysis of Western Blotting gray values of p-AKT/AKT, p-p130cas/p130cas, ALP, and RUNX2. Data were analyzed using one-way ANOVA, (*) p < 0.05 indicates a significant difference. (G) Calcification nodules were tested by Alizarin Red S after being cultured under different treatments. (H) Semi-quantitation of calcification. Data were analyzed using one-way ANOVA, (*) p < 0.05 indicates a significant difference.

MK2206, one small molecule compound that specifically inhibits AKT1 phosphorylation, was used to block the osteogenic differentiation of VICs promoted by Gli1. Western blotting showed that P-AKT, ALP, and Runx2 expression were increased in the Gli1 overexpression group while they were significantly decreased in the Gli1+MK2206 group** (Fig. 6A)**. The statistical analysis of the western blotting bands showed that the difference in protein expression was statistically significant **(Fig. 6B)**. After culturing the cells *in vitro* under OM condition for 15 days, the calcification nodules were evaluated by Alizarin Red S. Different groups showed different results by Alizarin Red S **(Fig. 6C)**. Semi-quantification of the calcification demonstrated a significant change between the Gli1 group and the Gli1+MK2206 group** (Fig. 6D)**. Furthermore, the expression of the BCAR1 gene was silenced by si-RNA to reduce P130cas. Western blotting indicated that P-AKT, ALP, and Runx2 expression was significantly reduced due to the down-regulation of P130cas** (Fig. 6E and Fig. 6F)**. After being cultured in OM condition for 15 days, the calcification nodules tested by Alizarin Red S were also different** (Fig. 6G)**. The semi-quantification of Alizarin Red S indicated that the silencing of P130cas attenuated the calcification induced by Gli1 **(Fig. 6H)**.

### Gli1 was activated through the canonical and non-canonical Hedgehog signaling pathways in VICs

**Figure 7:**
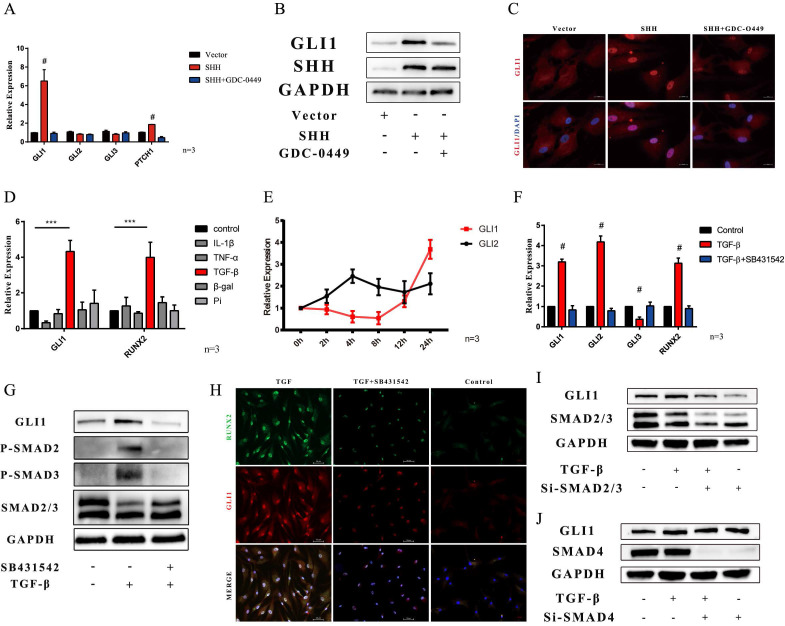
Gli1 activates through canonical and non-canonical Hedgehog signaling pathways in VICs. (A) PCR test of Gli1, Gli2, Gli3, Ptch1 genes in VICs after SHH treatment with or without GDC-0449. Data were analyzed using one-way ANOVA, (#) p < 0.05 indicates a significant difference. (B and C) Western blotting and immunofluorescence staining of Gli1 in VICs were consistent with gene expression. (D) PCR test of Gli1 and Runx2 gene expression in VICs treated by common calcification-inducing stimulators for 48h. Data were analyzed using one-way ANOVA, (* versus control) p < 0.05 indicates a significant difference. (E) Changes in Gli1 and Gli2 gene expression induced by tgf-β1 within 24 hours. (F) PCR test of Gli1, Gli2, Runx2 genes in VICs stimulated by tgf-β1 with or without SB431542. (G) Western blotting of p-SMAD2, p-SMAD3, SMAD2/3, and Gli1 protein in VICs stimulated by tgf-β1 with or without SB431542. (H) Immunofluorescence staining of Gli1 in VICs under different treatments. (I and J) Western blotting of Gli1 in VICs stimulated by tgf-β1 with si-SMAD2/3 or si-SMAD4.

The mechanism of up-regulation of Gli1 expression was also explored. Sonic hedgehog ligand is the triggered canonical hedgehog signaling pathway. The SHH gene was overexpressed in VICs. PCR analysis showed that Gli1, and PTCH1 gene increased in SHH overexpression group, while they were down-regulated in the SHH+GDC-0449 group, in which GDC-0449 is one specific small molecular hedgehog inhibitor **(Fig. 7A)**. Western blotting and immunofluorescence staining also confirmed that SHH induced Gli1 expression **(Fig. 7B and Fig. 7C)**. A non-canonical hedgehog signaling pathway is another possible mechanism that may happen in VICs. First, common calcification-inducing stimulators were used on VICs, and then, Gli1 and Runx2 gene expression was measured after 48 h by PCR. Gli1 gene along with RUNX2 gene was significantly increased after the induction with TGF-β1** (Fig. 7D)**. Then, the changes in Gli1 and Gli2 gene expression induced by TGF-β1 were evaluated within 24 hours, Gli1 gene increase started at 12 hours **(Fig. 7E)**. SB431542 is a classical inhibitor of the TGF-β type I receptor. PCR analysis indicated that Gli1, Gli2, and Runx2 genes significantly increased in the TGF-β1 group, while SB431542 could reverse this up-regulation induced by TGF-β1 **(Fig. 7F)**. Western blotting and immunofluorescence staining also confirmed that Gli1 protein increased after the stimulation with TGF-β, while it was inhibited by SB431542 **(Fig. 7G and Fig. 7H)**. Smad 2/3 factor was found phosphorylated during this biological process **(Fig. 7G)**. Next, smad2/3 factor was silenced by si-RNA, and western blotting indicated that Gli1 did not increase after the stimulation with TGF-β1** (Fig. 7I)**. Furthermore, smad4, which plays a key role in nuclear transportation in the TGF-β1 signaling pathway was down-regulated using si-RNA. Western blotting indicated that the silencing of smad4 did not influence Gli1 expression **(Fig. 7J)**.

## Discussion

Our research demonstrated that Gli1 promoted the calcification of VICs. Transcriptome sequencing analysis was used to explore the mechanism of this biological process. The transcriptome of VICs under different treatments was first sequenced. GO function annotation indicated that Gli1 enrichment regulated genes related to cell differentiation, inflammatory response, extracellular matrix organization, and positive regulation of cell proliferation. All these biological processes play critical roles during the pathological process of aortic valve calcification.

The present study separated the process of valve calcification into two different phases according to its histological features: fibrosis and biomineralization 24. The histological analysis revealed a significant thickening of the fibrotic layer, accompanied by a large degree of cell proliferation within the interstitial layer of the valve, resulting in a reduced elasticity and increased hardness of the valve leaflets. In contrast, normal human aortic valve interstitial cells were apparently in a resting situation, with a low viability, low proliferation, and low activity, to keep the valve soft and thin 25, 26. The overexpression of Gli1 significantly increased the viability and proliferation of VICs as revealed by CCK-8 assay, Edu, and Ki-67 staining. Flow cytometry cell cycle of VICs also indicated the regulation caused by the Gli1 factor. Western blotting and PCR showed that the cell cycle-related factor CCNB1 may play a key role in these changes. Most studies indicated that Gli1 directly increases the expression of CCND 27, but our study found a different result in VICs. Therefore, it is important to understand its deep mechanism.

Runx2 is one of the important factors that regulate the osteogenic differentiation of VICs 28, 29. When cells were cultured in OM, Gli1 accelerated the calcification of VICs by increasing the expression of Runx2. Further *ex-vivo* osteogenic differentiation model was performed. Tiny human aortic valve tissues were transfected with adenovirus. After two months under OM condition, various types of chemical staining were performed to show the calcification of the tissues. All these experiments showed the osteogenic function of the Gli1 factor. Interestingly, Co-IP experiments showed that Runx2 and Gli1 interacted with each other and cellular immunofluorescence staining also indicated this discovery. When the cells were treated with GANT61, Gli1 did not enter the nucleus, and the Gli1 and Runx2 complex also accumulated in the cytoplasm. Some research found that Runx2 directly regulates Gli1 and Ptch1 expression in osteoblast progenitors and osteoblasts 30. But our results indicated that Gli1 may also regulate Runx2 expression. As far as we know, this is the first time this phenomenon has been demonstrated in valve interstitial cells. It seems that Runx2 and hedgehog signaling regulates each other and induce osteogenic differentiation. Its internal mechanism is worthy of our in-depth exploration.

Gli1 is an important transcription factor in different biological processes. Therefore, ChIP-seq technology was performed in valve interstitial cells to explore the downstream target genes of the GLI1 transcription factor. Our results demonstrated that its TF binding motifs in VICs were CCACCC and TGGGTGG and 1478 genes related to the Gli1 transcription factor were up-regulated. The GO and KEGG enrichment analysis revealed that actin cytoskeleton, focal adhesion, and axon guidance were significantly activated. Furthermore, RNA-transcriptome sequencing was performed on three pairs of aortic valve tissues and the top ten significant signaling pathways were obtained by KEGG enrichment analysis. The interaction with the cellular transcriptome data revealed that the PI3K-AKT signaling pathway and focal adhesion signaling pathway were involved. Thus, an interactive analysis of two target gene sets was performed, revealing 15 genes as the final candidate genes. The protein P130cas encoded by BCAR1 is a member of the Crk-associated substrate (CAS) family of scaffold proteins, characterized by the presence of multiple protein-protein interaction domains. It has also many serine and tyrosine phosphorylation sites 31-33. Therefore, BCAR1 was selected as the final target gene that leads to the calcification of VICs caused by Gli1. However, the relationship between Gli1, AKT1, P130cas, and Runx2 needs further study.

AKT1 is the key intersection molecule of the two signaling pathways PI3K-AKT and focal adhesion signal pathway; thus, MK2206 and siRNA were used to regulate the expression of P-AKT1, P-P130cas, and P130cas. Our results showed the down-regulation of the osteogenic related proteins Runx2 and ALP in the Gli1 overexpression group when AKT1 phosphorylation was inhibited by MK2206. The osteogenic model *in vitro* also confirmed the osteogenic inhibitory effect, and the expression of P-P130cas was also reduced. These results revealed that the phosphorylation of AKT1 was the trigger of osteogenesis and phosphorylation of P130cas caused by Gli1. Our study found that P130cas was one of the target genes regulated by Gli1, which increased when the Gli1 factor was overexpressed. When P130cas expression was silenced by siRNA, the osteogenic related proteins ALP and Runx2 were also reduced although the phosphorylation of AKT1 was not affected. This result suggested that P130cas might play a key role in Gli1-induced calcification instead of AKT signaling pathway activation, which was involved in the extracellular matrix secretion when Gli1 was overexpressed in VICs. Consistent with this result, the cell morphology was significantly different in the Gli1 enrichment group compared to the control group., The pathophysiological process of valve tissue in CAVD involves the tissue remodeling: the cells of the valve tissue change, their number increases, and the extracellular matrix is remodeled 34. Our results showed that Gli1 was the transcription factor that changed all these three aspects of VICs.

The last part of our research was dedicated to evaluate the reason of Gli1 up-regulation in CAVD. Our focus was on the Hedgehog signaling pathway, since the core of this pathway lies in the Gli1 factor. Based on studies on this pathway over the past few decades, the mechanism of Gli1 upregulation in VICs was explored in two ways, such as through the activation of the canonical and non-canonical hedgehog signaling pathway 35. Interestingly, our results showed that both the two pathways activated the expression of the Gli factor in human aortic valve interstitial cells. TGF-β is one of the most significant activating factors of Gli transcription factors in VICs especially in the non-canonical pathway. TGF-β is one of the upstream molecules that are widely explored in the basic research of CAVD 12. A large number of studies showed that the increased expression of TGF-β in the aortic valve leads to the transformation of valve endothelial cells into VICs, remodels the extracellular matrix in valve tissues, and allows the osteogenic differentiation of VICs. The TGF-β-smad4 axis regulates the expression of the Gli1 factor, but our results revealed that smad4 did not affect the expression of Gli1 factor induced by TGF-β in VICs, while smad 2/3 factor was the regulator of Gli1 expression 36, 37. This is another interesting point that needs to be further explored.

## Supplementary Material

Supplementary tables.
