# Supplementary material for: Gli1 promotes the phenotypic transformation of valve interstitial cells through Hedgehog pathway activation exacerbating calcific aortic valve disease
Source: Int J Biol Sci. 2023 Apr 9;19(7):2053–66. doi: 10.7150/ijbs.74123 (PMC10158026; doi:10.7150/ijbs.74123)
Supplement: Supplementary file 1 — Supplementary tables. [file ijbsv19p2053s1.pdf]

Supplementary Table1:

Homo Runx2 F	GGCGGGTAACGATGAAAATT
Homo Runx2 R	GAGGCGGTCAGAGAACAAACTA
Homo GAPDH F	TCAAGAAGGTGGTGAAGCAGG
Homo GAPDH R	TCAAAGGTGGAGGAGTGGGT
Homo CCND1 F	GAAGATCGTCGCCACCTG
Homo CCND1 R	GACCTCCTCCTCGCACTTCT
Homo CCND2 F	CTGCTGGAATTGGTGTTGA
Homo CCND2 R	CTTCGTCTAGGAGCTGGAGG
Homo GLI1 F	ACATCAACAGCGAGCACATC
Homo GLI1 R	AGGTTTTCGAGGCGTGAGTA
Homo GLI2 F	TGAAGGATTCCTGCTCGTG
Homo GLI2 R	GAAGTTTTCCAGGACAGAACCA
Homo GLI3 F	AAGCGGTCCAAGATCAAGC
Homo GLI3 R	TGTTCCTTCCGGCTGTTC
Homo PTCH1 F	TGACAAAGCCGACTACATGC
Homo PTCH1 R	AGCGTACTCGATGGGCTCT
Homo CCNB1 F	ATGACATGGTGCACTTTCCTCC
Homo CCNB1 R	GCCACGTGCTGCATAACTGG
Homo CCNE1 F	AAATGGCCACAATCGACAGG
Homo CCNE1 R	CGAGGCTTGCACGTTGAGTT
Homo MKI67 F	GCCCCTGGAAGATTATGGTGG
Homo MKI67 R	GGGTTCTGACTGGTTGTGGTTGT

Primer sequences

Supplementary Table2

Protein	Brand	Cat. nub.
Gli1	Abcam	ab134906
ALP	R&D system	MAB1448
Runx2	CST.	8486
GAPDH	Proteintech	60004-1-Ig
Vimentin	Proteintech	10366-1-AP
α-SMA	Proteintech	14395-1-AP
Ki-67	Proteintech	CL594-27309
CyclinE1	Proteintech	11554-1-AP
CyclinB1	Proteintech	55004-1-AP
Runx2	CST.	12556
AKT	CST.	2920
p-AKT	CST.	4060
p130cas	CST.	13846
p-p130cas	CST.	4011
SHH	Abcam	ab240438
SMAD2/3	CST.	8685
SMAD4	CST.	46535
p-SMAD2	CST.	3108
p-SMAD3	CST.	9520

Antibodies
